# A Novel Score Combining Magnetic Resonance Spectroscopy Parameters and Systemic Immune-Inflammation Index Improves Prognosis Prediction in Non-Small Cell Lung Cancer Patients With Brain Metastases After Stereotactic Radiotherapy

**DOI:** 10.3389/fonc.2022.762230

**Published:** 2022-06-08

**Authors:** Dong Guo, Jiafeng Liu, Yanping Li, Qingqing Chen, Yunzheng Zhao, Xinwei Guo, Shuchai Zhu, Shengjun Ji

**Affiliations:** ^1^ Department of Radiation Oncology, Fourth Hospital of Hebei Medical University, Shijiazhuang, China; ^2^ Department of Radiotherapy, Rizhao Center Hospital, Rizhao, China; ^3^ Department of Radiation Oncology, Sunshine Union Hospital, Weifang, China; ^4^ Department of Radiotherapy & Oncology, The Affiliated Suzhou Hospital of Nanjing Medical University, Gusu School, Nanjing Medical University, Suzhou, China; ^5^ Department of Radiation Oncology, Affiliated Taixing People’s Hospital of Yangzhou University, Taixing, China

**Keywords:** NSCLC, brain metastases, SII, MRS, prognosis

## Abstract

**Objective:**

The aim of this study was to evaluate the prognostic significance of the combination of the magnetic resonance spectroscopy (MRS) parameters and systemic immune-inflammation index (SII) in patients with brain metastases (BMs) from non-small cell lung cancer (NSCLC) treated with stereotactic radiotherapy.

**Methods:**

A total of 118 NSCLC patients with BM who were treated with stereotactic radiotherapy were retrospectively enrolled in this study. All patients underwent MRS and blood samples test for SII analysis before the initiation of stereotactic radiotherapy. The correlation between the parameters of MRS and SII level was assessed using Spearman’s correlation coefficient. The cutoff values for the parameters of MRS, SII, and clinical laboratory variables were defined by the receiver operating characteristic (ROC) curve analysis to quantify these predictive values. The prognostic factors of overall survival (OS) and progression-free survival (PFS) curves were assessed using the Kaplan–Meier and Cox proportional hazards models.

**Results:**

The median follow-up time was 25 months (range, 12–49 months). The optimal cutoff point for the choline/creatine (Cho/Cr) ratio and SII were 1.50 and 480, respectively. The Cho/Cr ratio was negatively correlated with SII (rs = 0.164, *p* = 0.075), but there was a trend. The C-SII score was established by combining the Cho/Cr ratio and SII. Patients with both an elevated Cho/Cr ratio (>1.50) and an elevated SII (>480) were given a C-SII score of 2, and patients with one or neither were given a C-SII score of 1 or 0, respectively. The Kaplan–Meier analysis showed that a C-SII score of 2 was significantly linked with poor OS and PFS (*p* < 0.001 and *p* < 0.001, respectively). In the Cox proportional hazards model, the C-SII score independently predicted OS [hazard ratio (HR), 1.749; 95% CI, 1.176–2.601; *p* = 0.006] and PFS (HR, 2.472; 95% CI, 1.624–3.763; *p* < 0.001).

**Conclusion:**

The C-SII score was more accurate for predicting the clinical outcomes of NSCLC patients with BM who underwent stereotactic radiotherapy. The C-SII score, which was superior to either score alone, could be used to identify BM in NSCLC patients with poor outcomes.

## Introduction

Lung cancer is the most common cancer and the leading cause of cancer-related mortality in China (1). Non-small cell lung cancer (NSCLC) accounts for approximately 85% of all lung cancer cases, and NSCLC has a high predilection to metastasize to the brain (2). Brain metastasis (BM) occurs in approximately 20%–40% of patients with NSCLC and has become an important factor affecting the clinical outcomes of patients despite active treatments (3, 4). Radiation therapy includes stereotactic radiotherapy and whole-brain radiation treatment. Stereotactic radiotherapy has shown advantages over whole-brain radiotherapy as an effective treatment of choice for BM patients because of excellent local control and minimal toxicity (5–9). However, the patients’ median survival time is <12 months, and the quality of life of patients with BM is low, which poses a major challenge for NSCLC patients with BM (10). Reliable prognostic indicators must be identified to look for a better treatment choice and assess patient prognosis.

Consensus regarding abnormal metabolism in tumor tissue has been reached, and this study of metabolomics continues to provide new insight into the biological behavior of the tumor (11). Advanced magnetic resonance spectroscopy (MRS) technique can be applied to answer various clinical questions and evaluate cellular metabolic biochemical composition. The MRS technique provides principal metabolic biochemical composition such as *N*-acetyl-aspartate (NAA), choline (Cho), and creatine (Cr). Cho reflects synthesis and metabolism of cell membranes and turnover of the cell membrane during breakdown (12). Cr reflects the energy metabolism of the brain tissue cell, which are constant in normal brain tissue (13). NAA, a brain tissue neuronal marker, is reduced in the development of brain tumors (13). MRS parameters have widely been shown to be capable of identifying the biology of tumor metabolism and can monitor the treatment effect or tumor progression (14, 15). Our primary study has shown that the Cho/Cr ratio has been independently verified as a useful predictor in the prognosis of NSCLC patients (16). However, the occurrence of tumors is a comprehensive result of various systemic disorders, and its characteristics cannot be accurately predicted simply from one aspect. Cancer-related inflammation is a necessary component of the tumor microenvironment, and inflammatory cells may play a critical role in the development of malignancies (17). Furthermore, systemic immune-inflammation involves metabolic biochemical mechanisms where cancer cells express immune-inflammatory cytokines, potentially reflecting the biological activity of tumor cells. These theories continue to enhance our understanding of tumors and inflammation. In the clinics, systemic immune-inflammation index (SII), derived from neutrophil (N), lymphocyte (L), and platelet (P) in peripheral blood, has been deeply investigated in many malignancies. SII has become a reliable biomarker to reflect the immune and inflammation status of host and has been used as a prognostic index in multiple malignant cancers, including NSCLC (18–22). MRS detects the biological activity of cellular metabolism, while SII indicates immune-inflammation status, and both have been independently verified as useful predictors in the prognosis of NSCLC patients. However, clinical data focusing on the predictive value of combination of the MRS and SII for BM in NSCLC patients treated with stereotactic radiotherapy remain unknown.

This study was performed to explore the prognostic value of the C-SII score for BM in NSCLC patients treated with stereotactic radiotherapy and aimed to provide appropriate and individualized therapy in clinical treatment.

## Patients and Methods

### Study Design

**Figure 1:**
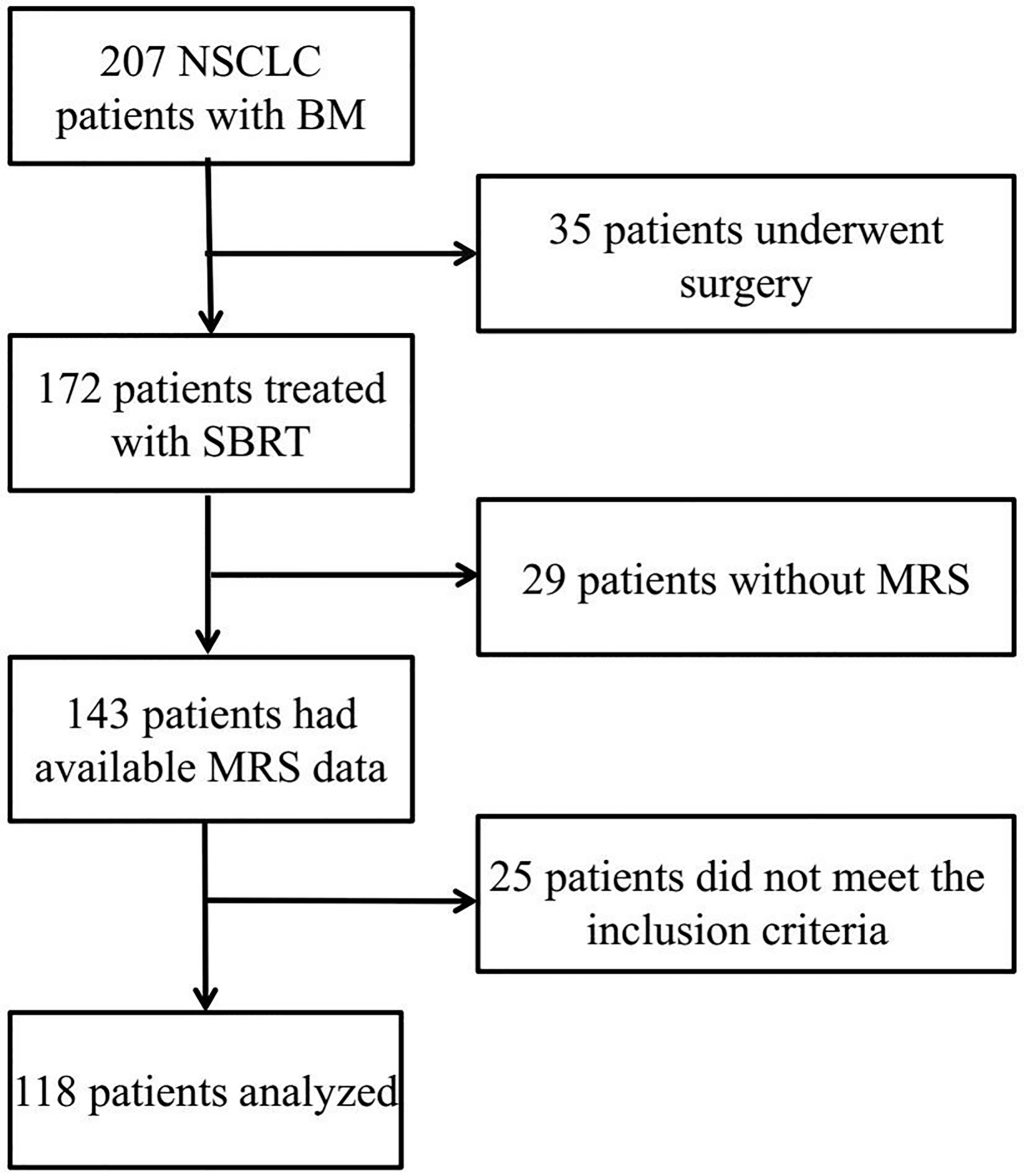
Patients’ screening process and results.

Between January 2014 and December 2017, the 118 patients with histologically confirmed NSCLC receiving stereotactic radiotherapy at The Affiliated Suzhou Hospital of Nanjing Medical University were retrospectively reviewed. Clinicopathological characteristics such as age, gender, Karnofsky Performance Status (KPS), patient histology, number of BMs, and TNM stage of patients were collected through their medical records. The primary lesions had been resected or stably controlled. The MRS metabolism composition and immune-inflammatory parameters were obtained from the peripheral blood and MRS, respectively. The inclusion criteria were as follows: i) age > 18 years, ii) biopsy histological confirmation of NSCLC, iii) Eastern Cooperative Oncology Group (ECOG) scores ranging from 0 to 2, iv) MRS imaging obtained before treatment with no contraindications, v) the number of BM ≤ 3, and vi) no hematological disorders. The specific patient flowchart is shown in **Figure 1**.

### Radiation Treatment

**Figure 2:**
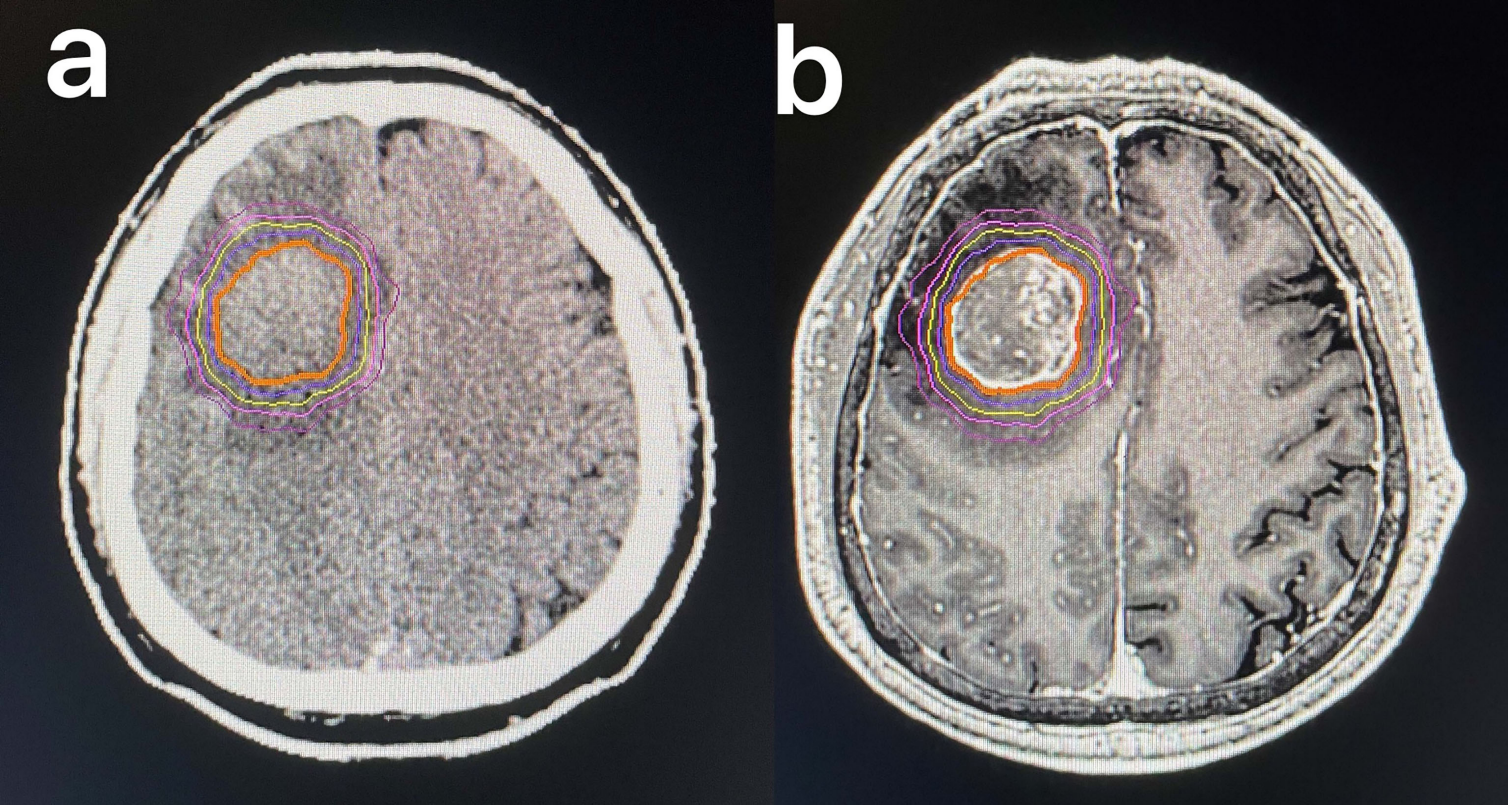
SBRT target area in a 58-year-old man with squamous cell lung cancer of the right frontal lobe: CT **(A)** and MRI **(B)**. SBRT, stereotactic body radiotherapy.

Enhanced CT scans (Philips Brilliance Big Bore CT) imaging was acquired and fused with a T1-weighted post-gadolinium MRI scan within 7 days of CT localization. The gross tumor volume (GTV) is accurately delineated by the identification of fusion images on the planning system, or ^18^F-fluorodeoxyglucose (FDG) PET/CT. At the same time, we have mapped out the critical organ structures (organ at risk (OAR)), including the brain stem, left eye, right eye, left optic nerve, right optic nerve, lens, and optic chiasm. The range of stereotactic radiotherapy doses used was 40–60 Gy. All radiation plans were created with arcs of 6-MV photons (**Figure 2**). Patients were given corresponding dehydration therapy to reduce brain edema during treatment.

### Magnetic Resonance Spectroscopy Analysis

**Figure 3:**
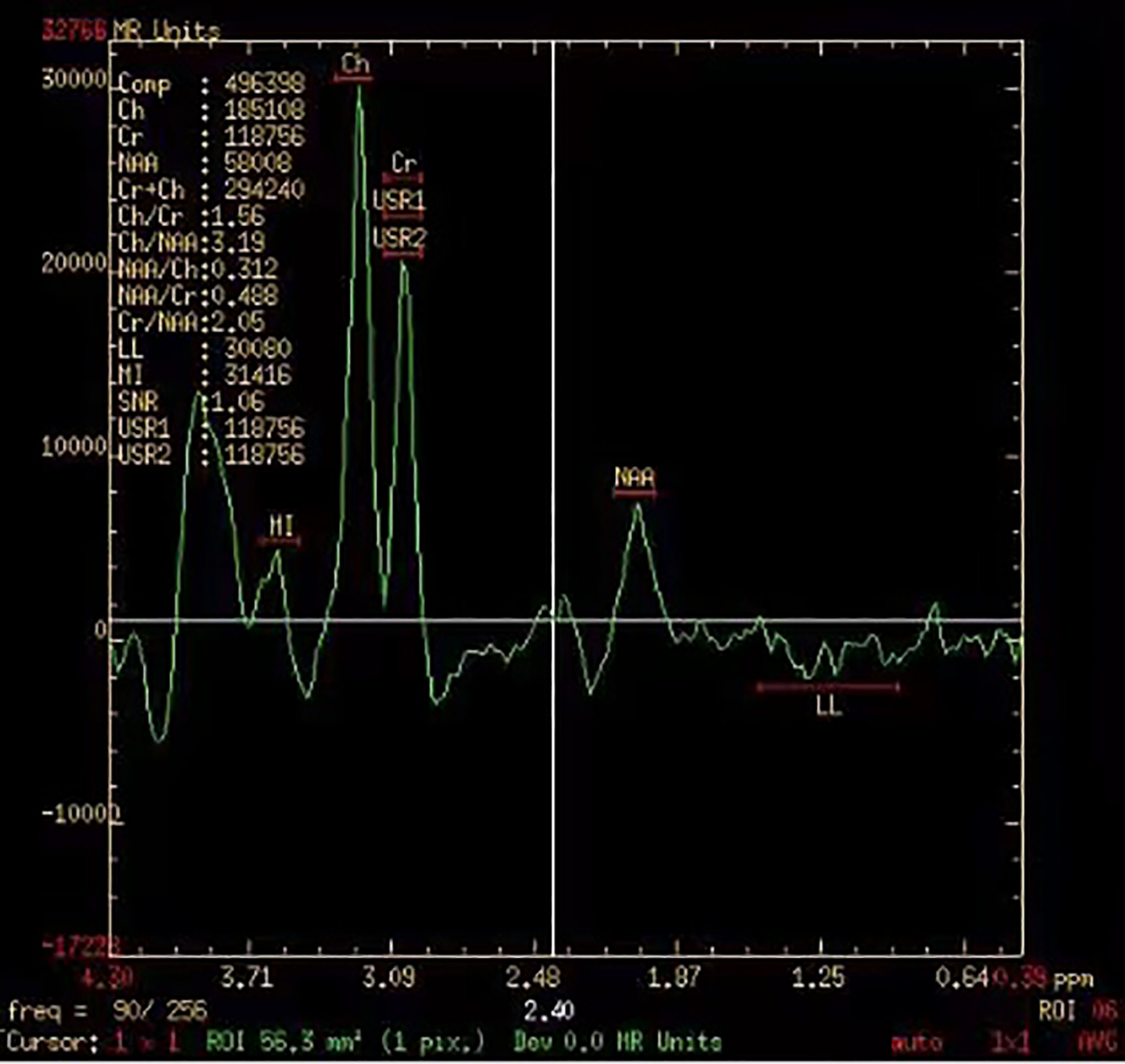
Magnetic resonance spectroscopy imaging of a 66-year-old women with NSCLC. NSCLC, non-small cell lung cancer.

The brain scan sequence (MRI plain scanning, enhanced MRI scan, and MRS scan) was performed using a 3.0-T clinical scanner magnetic resonance machine (Philips Healthcare, Andover, MA, USA). We used point-resolved spectroscopic (PRESS) to perform multivoxel MRS sequence scanning and determined the region of interest (ROI) by three clinicians and radiologists. Normal brain tissue at the opposite side of the tumor lesion was selected as the reference area. ^1^H-MRS scanning parameters were as follows: repetition time (TR), 1,500 ms; echo time (TE), 135 ms; field of view (FOV), 230 mm × 230 mm; slice thickness, 5 mm; scan time, 350 s; and voxel size, 1 × 1 × 1 mm^3^ (**Figure 3**). The MRS analysis software was used to automatically measure and calculate the relative metabolite intensities of the signals. MRS evaluation indicators included Cho, Cr, NAA, Cho/Cr ratio, and Cho/NAA.

### Peripheral Blood Sample Analysis

We collected peripheral venous blood from the patients 7 days before stereotactic radiotherapy. We strictly followed the inclusion criteria and performed related index analyses. SII was calculated using the formula SII = P * N/L, which is based on platelet (P), neutrophil (N), and lymphocyte (L) count. The neutrophil-to-lymphocyte ratio (NLR) was defined as the absolute neutrophil count divided by the absolute lymphocyte count. The platelet-to-lymphocyte ratio (PLR) was defined as the absolute platelet count divided by the absolute lymphocyte count.

### Definition of C-SII Score

Based on the optimal cutoff value, the Cho/Cr ratio and SII were split into low and high groups, respectively. In this study, we constructed a novel prognostic C-SII score system, which included both the Cho/Cr ratio and SII. We assigned a score of 0–2 to the Cho/Cr ratio (low and high) and SII (low and high). Patients were classified into three subgroups: low Cho/Cr ratio and low SII group (score = 0), low Cho/Cr ratio or low SII group (score = 1), and high Cho/Cr ratio and high SII group (score = 2).

### Statistical Analysis

The predictive value of the MRS and peripheral blood parameters was assessed by calculating the area under the ROC curve (AUC). A ROC curve was generated to obtain the Cho, Cr, NAA, Cho/Cr ratio, Cho/NAA, N, P, L, SII, NLR, and PLR cutoff values. Spearman’s coefficient test was performed to explore correlations between the Cho/Cr ratio and SII. Survival curves were analyzed to assess the survival time distribution by the Kaplan–Meier method and compared using the log-rank test to test the significance of overall survival (OS) and progression-free survival (PFS) among the different prognostic groups. Univariate and multivariate analysis logistic regression analyses were used to determine independent prognostic factors. Two-sided *p*-values <0.05 were considered statistically significant. All analyses were conducted by using SPSS v19.0 software (SPSS, Inc., Chicago, IL, USA) and GraphPad Prism 5 software (GraphPad, San Diego, CA, USA).

## Results

### Clinicopathological Characteristics of Patients

As shown in **Table 1**, a total of 118 patients were enrolled in this retrospective study. Ages ranged from 52 to 71 years (median, 59 years). Most patients (54.2%) were female, and the majority (74.6%) has adenocarcinoma. The findings showed 72 (61.0%) with 1 BM and 88 (74.6) patients with neurologic symptoms. Regarding staging, 75 patients (63.6%) had T1–T2 stage, and 31.4% of patients had N2 and N3 stage tumors. After a median follow-up of 25 months (range 15–49 months), 69 of the 118 (58.5%) patients suffered recurrence and metastasis, and 66 (55.9%) patients died.

**Table 1:** The clinical characteristics of all NSCLC patients with BM.

Variables	Values
Gender	
Male Female	54 (45.8) 64 (54.2)
Age (years)	
<60 ≥60	58 (49.2) 60 (50.8)
Smoke	
No Yes	53 (44.9) 65 (55.1)
KPS	
90-100 70-80	55 (46.6) 63 (53.4)
Patient histology	
SCC AD	30 (25.4) 88 (74.6)
Number of BMs	
1 2-3	72 (61.0) 46 39.0)
Maximum diameter	
< 2cm ≥2 cm	62 (52.5) 56 (47.5)
Neurologic symptoms	
No Yes	30 (25.4) 88 (74.6)
Tumor stage	
T1-T2 T3-T4	75 (63.6) 43 (36.4)
Node stage	
N0-N1 N2-N3	81 (68.6) 37 (31.4)
TNM stage	
I II-III	61 (51.7) 57 (48.3)
CEA	
Normal Elevated	70 (59.3) 48 (40.7)

KPS, Karnofsky performance score; SCC, squamous cell carcinoma; AD, adenocarcinoma; BM, brain metastases; CEA, carcinoembryonic antigen.

### Choline/Creatine Ratio and Systemic Immune-Inflammation Index

**Figure 4:**
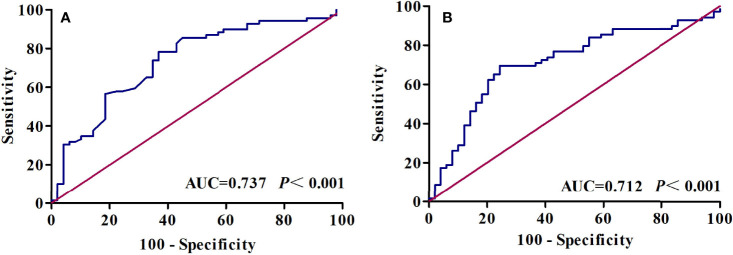
Receiver operating characteristic (ROC) curve analysis of **(A)** Cho/Cr and **(B)** SII. Cho, choline; Cr, creatine; SII, systemic immune-inflammation index.

**Figure 5:**
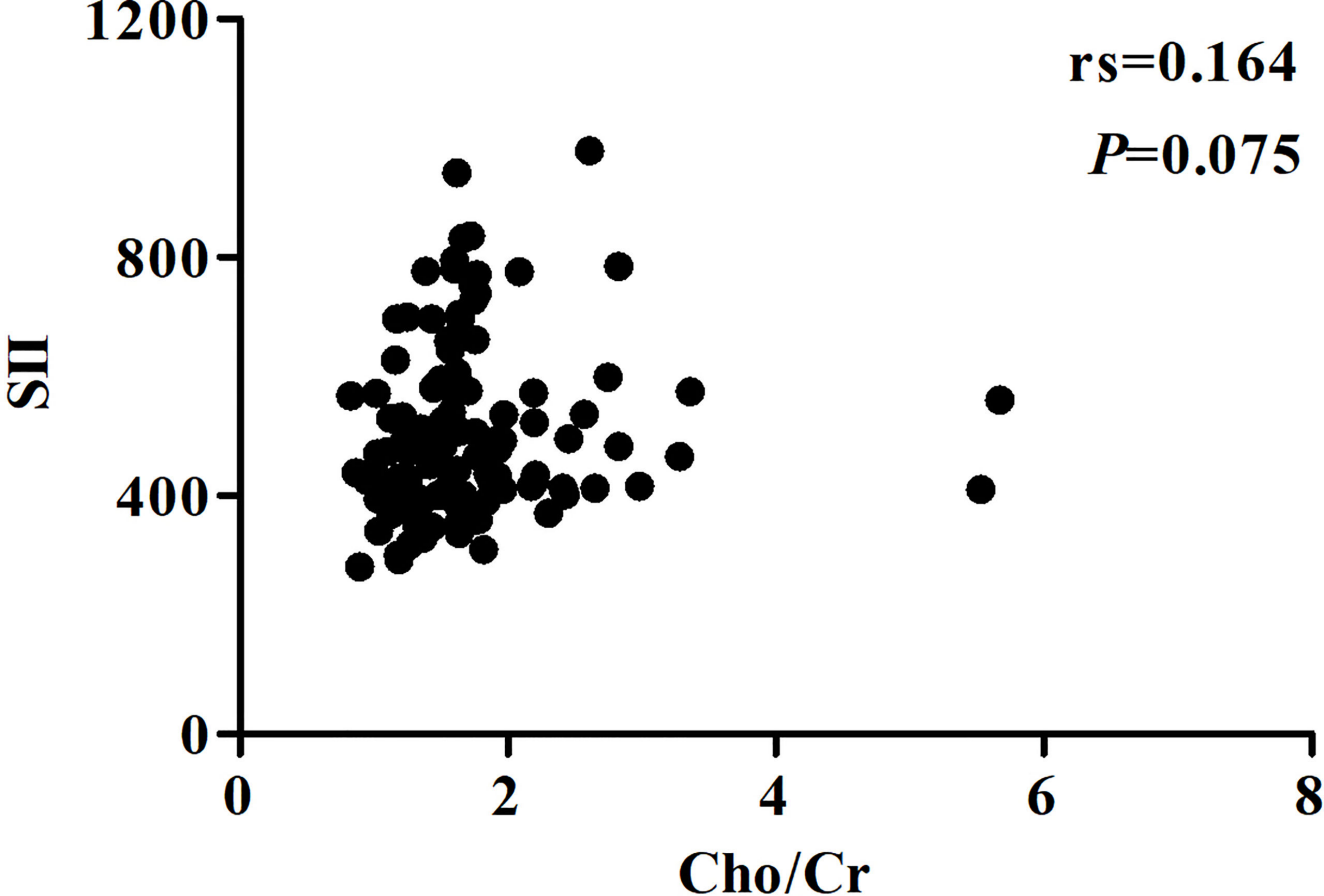
Correlations between Cho/Cr and SII. Cho, choline; Cr, creatine; SII, systemic immune-inflammation index.

With Youden’s J-statistics establishing 1.50 (sensitivity, 78.3%; specificity, 61.2%) as optimal cutoff values for the Cho/Cr ratio to predict PFS, the ROC analysis calculated the AUC as 0.737. The same analysis showed that the optimal cutoff value was 480 (sensitivity, 69.6%; specificity, 75.5%) for SII with the largest AUC of 0.712 (**Figure 4**). The Cho/Cr ratio and SII levels of pretreatment were 1.60 (0.83–5.68) and 481.71 (280.29–978.81), respectively. **Table 2** presents the results of quantitative parameters of MRS and immune-inflammatory. We observed a negative correlation between the Cho/Cr ratio and SII, but there was a positive trend (*p* = 0.075; Spearman’s correlation coefficient, rs = 0.164) (**Figure 5**).

**Table 2:** A summary of MRS metabolic characteristics and inflammation index.

	Median (range)
**Signal**	
Cho	2.03 (0.93-3.37)
Cr	1.28 (0.43-2.20)
NAA	0.82 (0.21-1.72)
Cho/Cr	1.60 (0.83-5.68)
Cho/NAA	2.44 (0.89-14.38)
**Peripheral blood inflammation index**	
Neutrophil	4.91 (2.36-9.88)
Lymphocyte	1.93 (0.82-3.57)
Platelet	188 (102-402)
SII	481.71 (280.29-978.81)
NLR	2.59 (1.37-5.97)
PLR	99.21 (38.98-168.00)

Cho, choline; Cr, creatine; NAA, N-acetyl-aspartate; NLR, Neutrophil-to-lymphocyte ratio; PLR, Platelet-to-lymphocyte ratio; SII, Systemic immune-inflammation index.

### Survival Analysis

**Figure 6:**
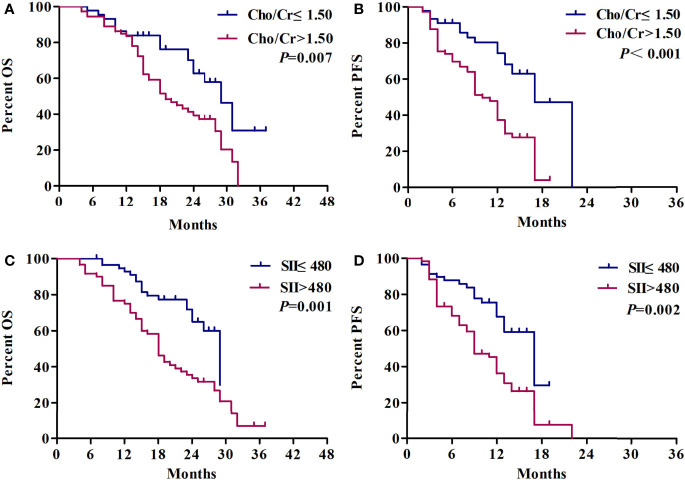
Kaplan–Meier survival curves of Cho/Cr and SII in NSCLC patients with brain metastases. **(A)** Overall survival curves of Cho/Cr. **(B)** Progression-free survival curves of Cho/Cr. **(C)** Overall survival curves of SII. **(D)** Progression-free survival curves of SII. Cho, choline; Cr, creatine; SII, systemic immune-inflammation index.

**Figure 7:**
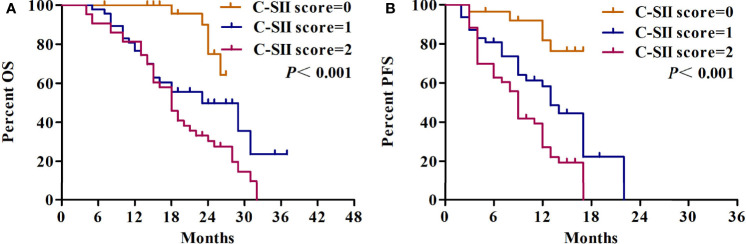
Kaplan–Meier survival curves depicting outcomes of overall survival **(A)** and progression-free survival **(B)** according to the C-SII score.

After a median follow-up of 25 months, OS (median OS 23 *vs.* 18 months) and PFS (median PFS 13 *vs.* 9 months) in the low Cho/Cr ratio group as compared with the high Cho/Cr ratio were significantly prolonged. In particular, patients with high SII showed a shorter OS (median OS 18 *vs.* 20 months) and PFS (median PFS 9 *vs.* 11.5 months) (**Figure 6**). The median OS in patients with a C-SII score of 2 was significantly lower than the OS in patients with a C-SII score of 1 and a C-SII score of 0 (18 vs. 18 vs. 23 months; *p* < 0.001) (**Figure 7A**). The median PFS rates were 13, 9, and 9 months for patients with C-SII scores of 0, 1, and 2, respectively (*p* < 0.001) (**Figure 7B**).

Univariate and multivariate Cox regression analyses were performed to explore the factors affecting survival in this study. The univariate analysis revealed that age (*p* = 0.022), smoking status (*p* = 0.023), and KPS (*p* = 0.003) were significantly associated with OS; KPS (*p* = 0.014) and patient histology (*p* = 0.003) were significantly associated with PFS. In terms of MRS and peripheral blood parameters, PLR (*p* = 0.033, *p* = 0.006), SII (*p* = 0.001, *p* = 0.002), and Cho/Cr ratio (*p* = 0.007, *p* < 0.001) were positively associated with OS and PFS. We found no significant association of survival with the number of BMs, maximum diameter, neurologic symptoms, and TNM stage (**Tables 3**, **4**).

**Table 3:** Univariate analysis of OS and PFS based on clinical-pathological data.

Variables	OS			PFS		
	HR	95% CI	P-value	HR	95% CI	P-value
Gender						
Male Female	Reference 0.911	0.560-1.482	0.707	Reference 0.929	0.577-1.495	0.761
Age (years)						
<60 ≥60	Reference 1.791	1.088-2.946	0.022	Reference 1.476	0.909-2.386	0.115
Smoke						
No Yes	Reference 1.847	1.089-3.133	0.023	Reference 1.536	0.937-2.517	0.089
KPS						
90-100 70-80	Reference 2.206	1.298-3.750	0.003	Reference 1.874	1.133-3.099	0.014
Patient histology						
SCC AD	Reference 1.294	0.717-2.337	0.392	Reference 2.797	1.418-5.519	0.003
Number of BMs						
1 2-3	Reference 0.828	0.501-1.371	0.464	Reference 0.879	0.536-1.441	0.608
Maximum diameter						
< 2cm ≥2 cm	Reference 0.976	0.602-1.582	0.920	Reference 1.264	0.784-2.039	0.336
Neurologic symptoms						
No Yes	Reference 0.733	0.449-1.196	0.213	Reference 0.868	0.538-1.400	0.562
Tumor stage						
T1-T2 T3-T4	Reference 0.604	0.349-1.047	0.072	Reference 0.834	0.501-1.388	0.484
Node stage						
N0-N1 N2-N3	Reference 0.782	0.449-1.362	0.385	Reference 1.077	0.642-1.806	0.778
TNM stage						
I II-III	Reference 1.048	0.639-1.718	0.852	Reference 1.136	0.704-1.834	0.602
CEA						
Normal Elevated	Reference 0.879	0.535-1.444	0.611	Reference 1.158	0.717-1.870	0.548

OS, overall survival; PFS, progression-free survival; HR, hazard ratio; CI, confidence interval; KPS, Karnofsky performance score; SCC, squamous cell carcinoma; AD, adenocarcinoma; BM, brain metastases; CEA, carcinoembryonic antigen.

**Table 4:** Univariate analysis of OS and PFS based on MRS metabolic characteristics and inflammation index.

Variables	OS			PFS		
	HR	95% CI	P-value	HR	95% CI	P-value
Cho						
≤2.50 >2.50	Reference 1.223	0.696-2.150	0.484	Reference 1.551	0.902-2.665	0.113
Cr						
≤0.50 >0.50	Reference 0.641	0.155-2.648	0.539	Reference 0.501	0.121-2.074	0.341
NAA						
≤1.50 >1.50	Reference 0.896	0.325-2.472	0.832	Reference 1.188	0.477-2.960	0.712
Cho/Cr						
≤1.50 >1.50	Reference 2.140	1.230-3.723	0.007	Reference 2.863	1.586-5.169	<0.001
Cho/NAA						
≤3.50 >3.50	Reference 1.485	0.903-2.442	0.119	Reference 1.619	0.995-2.633	0.052
Neutrophil						
≤6.13 >6.13	Reference 1.682	0.993-2.849	0.053	1.581	0.935-2.671	0.087
Lymphocyte						
≤2.70 >2.70	Reference 1.507	0.717-3.170	0.280	Reference 1.441	0.727-2.858	0.296
Platelet						
≤169 >169	Reference 1.336	0.794-2.246	0.275	Reference 1.361	0.812-2.280	0.242
SII						
≤480 >480	Reference 2.513	1.445-4.370	0.001	Reference 2.297	1.370-3.851	0.002
NLR						
≤2.50 >2.50	Reference 1.296	0.769-2.187	0.330	Reference 1.182	0.724-1.929	0.503
PLR						
≤91.50 >91.50	Reference 1.854	1.053-3.265	0.033	Reference 2.211	1.261-3.876	0.006

OS, overall survival; PFS, progression-free survival; HR, hazard ratio; CI, confidence interval; Cho, choline; Cr, creatine; NAA, N-acetyl-aspartate; NLR: Neutrophil-to-lymphocyte ratio; PLR: Platelet-to-lymphocyte ratio; SII: Systemic immune-inflammation index.

To verify the predictive value of the C-SII score in NSCLC with BM, we performed multivariate Cox proportional hazards regression analysis including age, smoking status, KPS, patient histology, and C-SII score. The C-SII score is an independent prognostic factor for OS (hazard ratio (HR), 1.749; 95% CI, 1.176–2.601; *p* = 0.006) and PFS (HR, 2.472; 95% CI, 1.624–3.763; *p* < 0.001) (**Table 5**).

**Table 5:** Multivariate Cox regression analysis of OS and PFS.

Variables	OS			PFS		
	HR	95% CI	P-value	HR	95% CI	P-value
Age (years)						
<60 ≥60	Reference 1.749	1.028-2.978	0.039			
Smoke						
No Yes	Reference 1.106	0.618-1.980	0.735			
KPS						
90-100 70-80	Reference 1.515	0.848-2.706	0.161	Reference 1.263	0.746-2.139	0.385
Patient histology						
SCC AD				Reference 4.217	1.657-10.736	0.003
PLR						
≤91.50 >91.50	Reference 1.209	0.654-2.237	0.545	Reference 0.556	0.242-1.273	0.165
C-SII score						
0 1-2	Reference 1.749	1.176-2.601	0.006	Reference 2.472	1.624-3.763	<0.001

BM, brain metastases; HR, hazard ratio; CI, confidence interval; OS, overall survival; PFS, progression-free survival; Cho, choline; Cr, creatine; NAA, N-acetyl-aspartate.

## Discussion

A reliable prognostic prediction score system is crucial in risk stratification for patients and for adjusting appropriate treatment strategies for NSCLC with BM. In the present study, we investigated the utility of the Cho/Cr ratio and SII and C-SII scores on prognosis in NSCLC patients. The results showed that the C-SII score is an independent predictor of OS and PFS among NSCLC patients with BM.

In recent years, stereotactic radiotherapy is used to treat limited numbers of BM, since this therapy is less invasive than drugs and surgical resection and has better local control (23, 24). Therefore, the use of stereotactic radiotherapy is recommended to further control BM risk (25). At present, various blood indicators have been evaluated for their prognostic roles in NSCLC patients with BM, such as neuron‐specific enolase (NSE) (26), carcinoembryonic antigen (CEA) (27), and Lung-molGPA (28). However, there are no reliable predictors that can reflect different tumor biological behaviors. Hence, searching for accurate prognostic factors is of great clinical application value.

The occurrence of tumors is often accompanied by changes in metabolic biochemical composition. MRS is a non-invasive and sensitive imaging method that allows researchers to measure and visualize metabolic biochemical information from brain tumor tissues (29). Increasing evidence has indicated that MRS can identify tumor active regions and enhance more individualized response-based treatment in high-grade glioma (15). An ongoing effort at the Tehran University of Medical Sciences has shown that MRS parameters can improve the accuracy of predictive nomograms to assess the risk of biochemical recurrence after radical prostatectomy in prostate cancer (30). More comprehensive understanding of the biochemical composition changes in metabolites for tumors is urgently needed. The typical MRS metabolic abnormalities of BM often include increased Cho and decreased NAA and Cr. Minicozzi et al. observed in thirty-six head and neck cancer cases that the Cho/Cr ratio is significantly elevated in the group with poor response (14). Fink et al. found that multivoxel MRS Cho/Cr ratio peak-area shows a great advantage for distinguishing glioma recurrence (31). Negendank and colleagues conducted a co-operative study with 15 clinical research centers and confirmed that Cho was higher in glial tumors than in non-involved brain tissues (32). Dowling and coworkers revealed that Cho concentrations and NAA in tumor tissue were higher than normal values cancer (33). In this study, our result reported that the Cho/Cr ratio was an independent relevant factor for death and progression (*p* = 0.006 and *p* < 0.001). Whether Cho and Cr were not correlated with prognosis of NSCLC with BM is unknown.

Accumulating studies have substantiated that peripheral venous blood markers can reflect the condition of the host immune-inflammation status. Counts of the peripheral immune-inflammatory cells, such as platelet, lymphocytes, and neutrophils, have confirmed the reliable association link between inflammatory cells and prognosis in malignant tumors (34–37). SII is an integrated parameter, including platelets, neutrophils, and lymphocytes, and has been proved to be an independent predictor of malignant tumors (22, 38–43). The value of SII in predicting clinical outcomes for cancer patients may be associated with the function of platelets, neutrophils, and lymphocytes. Platelets release growth factors and pro-angiogenic protein and protect tumor cells from an immune response (44). Neutrophils can take part in various stages of growth and metastasis of tumors and generate immunosuppressive effects by producing and secreting cytokines, chemokines, and proteases (45). In contrast to neutrophils, lymphocytes exert an important antitumor immune response and have been proved to be related to systematic immune surveillance (46). In this study, the association between SII and clinical outcomes of patients in NSCLC with BM was evaluated. Our results indicated that SII was significantly associated with OS and PFS (*p* = 0.001 and *p* = 0.002).

Clinicians can evaluate clinical indicators such as tumor size, degree of tumor differentiation, or tumor location, but these evaluation criteria are based on individual subjective evaluation and judgment. The heterogeneity of individual tumors is largely reflected in the biological characteristics of tumors and the host immune-inflammatory state. Considering a problem from multiple angles can lead to breakthroughs. Recently, many scholars have realized that combining two peripheral blood indexes can be considered a useful independent prognostic marker of tumors. In the retrospective study initiated by Chen et al., their results revealed that the combination of circulating tumor cells with CEA has a better disease prediction than alone in NSCLC patients (47). Huang et al. showed that preoperative combined NLR and fibrinogen concentration can be used as independent prognostic indicators for OS (HR, 1.512; 95% CI, 1.283–1.783; *p* < 0.001) (48). Although Guo et al. (49) and Schernberg et al. (50) conducted related studies on PET/CT combined with blood inflammation indicators predicting prognosis and obtained positive results, clinical data focusing on the predictive value of the combination of the MRS and SII for BM in NSCLC patients treated with stereotactic radiotherapy have not been reported. We previously used MRS alone to evaluate the prognosis of patients with BM in NSCLC patients and revealed that a positive Cho/Cr ratio was an independent risk factor for OS (*p* = 0.009) and PFS (*p* = 0.006) (16). The Cho/Cr ratio or SII alone may not be sufficient to accurately reflect the tumor characteristics. Using the C-SII scores system may be a more accurate choice. In our study, our results revealed that patients with a C-SII score of 2 (Cho/Cr > 1.50 and SII > 480) have poorer clinical outcomes than patients with a C-SII score of 1 (Cho/Cr > 1.50 or SII > 480) or C-SII score of 0 (Cho/Cr ≤ 1.50 and SII ≤ 480). In the multivariate Cox regression analyses, study results demonstrated that the C-SII score independently predicted OS (HR, 1.749; 95% CI, 1.176–2.601; *p* = 0.006) and PFS (HR, 2.472; 95% CI, 1.624–3.763; *p* < 0.001).

We established a C-SII score system by combining the Cho/Cr ratio and SII in this study, and preliminary results showed that it was an accurate and reliable system for evaluating prognosis in NSCLC patients with BM. However, only 118 NSCLC with BM patients were assessed in this study because of the limited number of enrolled cases. In addition, incomplete clinical data and loss of follow-up were inevitable because of the long duration of this retrospective study. There is selection bias when clinicians and radiologists use MRS to determine the ROI. These limitations require further evaluation, and we need a validation cohort to further verify our conclusions in the future.

## Conclusion

NSCLC patients with a high Cho/Cr ratio and high SII had significantly poor outcomes in the present study. The C-SII score system is a strongly unfavorable survival index in assessing risk stratification for NSCLC patients with BM, which suggests that clinicians should adjust the treatment strategy and generalize clinical application.

## Data Availability Statement

The original contributions presented in the study are included in the article/supplementary material. Further inquiries can be directed to the corresponding author.

## Ethics Statement

This study was approved by the ethics committee of the Rizhao Center Hospital and The Affiliated Suzhou Hospital of Nanjing Medical University. This study was conducted in strict accordance with the national institutes of health guidelines. The patients/participants provided their written informed consent to participate in this study. Written informed consent was obtained from the individual(s) for the publication of any potentially identifiable images or data included in this article.

## Author Contributions

DG, JL, YL, QC, YZ, XG, SJ, and SZ prepared and reviewed the manuscript. All authors contributed to the article and approved the submitted version.

## Funding

This study was supported by a grant (GSWS2020067) from the Gusu Health Talent Program.

## Conflict of Interest

The authors declare that the research was conducted in the absence of any commercial or financial relationships that could be construed as a potential conflict of interest.

## Publisher’s Note

All claims expressed in this article are solely those of the authors and do not necessarily represent those of their affiliated organizations, or those of the publisher, the editors and the reviewers. Any product that may be evaluated in this article, or claim that may be made by its manufacturer, is not guaranteed or endorsed by the publisher.
